# Development of a Near Ground Remote Sensing System

**DOI:** 10.3390/s16050648

**Published:** 2016-05-06

**Authors:** Yanchao Zhang, Yuzhao Xiao, Zaichun Zhuang, Liping Zhou, Fei Liu, Yong He

**Affiliations:** College of Biosystems Engineering and Food Science, Zhejiang University, Hangzhou 310058, China; yczhang@zju.edu.cn (Y.Z.); yzxiao@zju.edu.cn (Y.X.); 21313019@zju.edu.cn (Z.Z.); 21413023@zju.edu.cn (L.Z.); fliu@zju.edu.cn (F.L.)

**Keywords:** UAV, aerial imaging, simulation platform, slideways

## Abstract

Unmanned Aerial Vehicles (UAVs) have shown great potential in agriculture and are increasingly being developed for agricultural use. There are still a lot of experiments that need to be done to improve their performance and explore new uses, but experiments using UAVs are limited by many conditions like weather and location and the time it takes to prepare for a flight. To promote UAV remote sensing, a near ground remote sensing platform was developed. This platform consists of three major parts: (1) mechanical structures like a horizontal rail, vertical cylinder, and three axes gimbal; (2) power supply and control parts; (3) onboard application components. This platform covers five degrees of freedom (DOFs): horizontal, vertical, pitch, roll, yaw. A stm32 ARM single chip was used as the controller of the whole platform and another stm32 MCU was used to stabilize the gimbal. The gimbal stabilizer communicates with the main controller via a CAN bus. A multispectral camera was mounted on the gimbal. Software written in C++ language was developed as the graphical user interface. Operating parameters were set via this software and the working status was displayed in this software. To test how well the system works, a laser distance meter was used to measure the slide rail’s repeat accuracy. A 3-axis vibration analyzer was used to test the system stability. Test results show that the horizontal repeat accuracy was less than 2 mm; vertical repeat accuracy was less than 1 mm; vibration was less than 2 g and remained at an acceptable level. This system has high accuracy and stability and can therefore be used for various near ground remote sensing studies.

## 1. Introduction

UAVs have been increasingly used in agriculture for remote sensing. They can fly over different types of landscape and image farmland on a large scale in a short time. Compared to satellite remote sensing and manned aircraft remote sensing, UAV remote sensing has several advantages: (1) acquisition of high resolution aerial photos, as most times UAVs fly under 1000 m and can achieve high spatial resolution [1] which is enough for field spatial variability observation; (2) when insects or plant blast disease come on suddenly, UAVs can respond in a short time, while satellites only overfly the same spot periodically; (3) UAVs require small take-off and landing fields—multirotor [2] and helicopter [3] versions take off and land vertically, while fix-wing [4] ones can take off via ejection and land via parachute, so UAVs don’t require reserved ground fields like airports or take-off runways like remote sensing aircraft. Moreover, thanks to whole UAV industry booming, cost of UAV is decreasing significantly. All the above advantages show that UAVs have great potential in many aspects. They help farmers survey their farmland and keep them informed about what is happening on their farmland. In recent years, more and more researchers have used UAVs for remote sensing and monitoring experiments, like tropical forest monitoring [5], coastal area monitoring [6], monitoring of erosion of rill and interrill [7], geological mine site mapping [8], infield crop monitoring [9], *etc*.

As UAVs are increasingly used for remote sensing, more types of sensors are being modified for or developed to be mounted on UAVs. Imaging sensors are a crucial part of remote sensing platforms like satellites, aircraft, UAVs and balloons. Sensors mounted on UAVs should satisfy the following requirements: (1) Sensors must be lightweight and small-size, the limited UAV payload and cargo space size being the main reason. Usually flight authorities place restrictions on the take-off weight for UAVs, like the U.S. FAA which has strict restrictions on UAVs heavier than 25 kg. Large and heavy sensors must be modified to fit UAVs; (2) Sensors can record data automatically or by remote control. Existing literature shows that DSLR [10], Tetracam adc [11] and MCA6 [12,13], thermal cameras [14], hyperspectral imaging sensors [15,16] and lidar [17] are now available on UAVs; (3) Sensors can resist UAV waggle by itself or by post-processing the image data. Waggle is inevitable in UAV remote sensing. For planar imaging sensors like DSLR cameras or ADC cameras, the waggle will blur pictures and make image post-processing difficult. For pushbroom cameras like hyperspectral imaging sensors, it’s crucial to tackle with UAV waggle in the air. In summary, even before the remote sensor is mounted on the UAV, it needs to be tested to verify if it can work properly. UAV-related photography methods and image analysis theory are developing, like automated georeferencing [18], object-based image analysis [19], image registration [20], time-series analysis [21], object-based image segmentation [22] and more using methods are to be discovered. There are a lot of experiments to do to develop new sensors and find better ways to use UAVs.

For now, UAV aren’t as reliable as other platforms. A successful UAV outdoor flight involves many factors. Unexpected or unrecognized reasons may lead to flight failure, like RC signal loss, strong winds, operator errors, FC malfunctions, *etc*. Errors in maintenance may also lead to flight failure. Compared to the UAVs themselves, the sensors mounted on them are mostly more expensive, so researchers are often reluctant to do experiments. Moreover, outdoor flight is limited by the weather conditions. UAV can’t fly on rainy days and are not supposed to fly against instantaneous wind speeds above 10 m/s. Cloud and fog are non-ignorable factors. They will degrade remote sensing image quality and cause shade and reflectance non-uniformity. It is a huge challenge to acquire surface features at high resolution under the influence of shadows [23]. Mõttus [24] quantified blue sky radiation caused by scattering in the atmosphere and showed that shadow-caused scattering has a great influence on canopy PRI. Weather may limit UAV research which involves periodical experiments. UAV systems are highly sophisticated and requires both manufacturers and operators to have a profound knowledge of radio communication, electric circuits, engine principles, control theory, structures, materials and meteorology. Since a standard system hasn’t been established for industrial UAV manufacturers, UAVs in use are likely to have unidentified malfunctions. As well human errors can hardly be eliminated, so there is a chance that UAVs will crash. If crashes happen, researchers will suffer a great loss and may have to suspend their research projects.

Controlled remote sensing conditions are necessary to verify whether newly proposed remote sensing processing methods for UAVs are correct. Before developing a UAV remote sensing system, it is necessary to theoretically test if the sensor system can achieve the research goals. Near ground remote sensing is the preparation for UAV remote sensing and can avoid losses caused by unexpected UAV crashes. For now, remote sensing platforms mainly consist of satellite, airborne, UAV, mobile platforms, and static platform [25]. All these platforms sense the Earth in an arbitrary environment. Some remote sensing theories like how haze degrades the remote sensing data [26] or what information reflectance light carries at different angle [27,28] need to be verified in controlled environments. To build a more precise model, controlled environment experiments are necessary to find out how the different factors correlate. UAV remote sensing is new compared to other platforms. It can work at lower altitude and obtain finer information. New theories which are different from traditional ways are emerging as well. To develop remote sensing models from empirical, subjective, crude experience, a near ground remote sensing system is needed for detailed research, yet there are few works about the establishment of in-laboratory near ground remote sensing platforms.

To make UAV remote sensing motes practical, a near ground test platform is necessary. The aim of this research was to develop a highly integrated remote sensing simulation platform for imaging sensor testing and ground-based remote sensing. This system can move in five DOFs including horizontal, vertical, pan, tilt and roll. It resembles how UAV remote sensing works. This platform can be divided into three parts: machinery components, electrical controller and GUI software. Linear slideways were used for horizontal motion and an actuator was used for vertical motion. Servo motors were used to drive the system. A central controller was developed to control the servo motors and far-end gimbal stabilizer. A CAN bus was used to connect the far-end gimbal stabilizer with the central controller via a 20 m long cable. At the far end, a gimbal controller was developed to stabilize the main gimbal. Stabilizing algorithms were written in the gimbal controller to maintain the gimbal at the desired altitude. GUI software run on a PC was developed for users to observe how the system is working and to set platform working parameters. Functionality tests were done to prove that this system is highly precise and stable. The horizontal repeated accuracy is less than 2 mm and the vertical is less than 1 mm. Peak vibrations are less than 2 g.

## 2. Materials and Methods

### 2.1. System Design

This system was to be built in a 12 × 3.5 m^2^ room on a rooftop of a seven floor building. The room is 3.7 m tall. It is located at N30.2983, E120.0909 in Hangzhou, China. The room ceiling is made of square steel tube as skeleton and fiberglass reinforced plastic as rain-proofing. The ceiling is strong enough to sustain the platform hung from it. This room is open to sunlight so that light conditions of this room are very good. Moreover, shade curtains were installed to meet the demands of some experiments which have particular light requirements.

**Figure 1:**
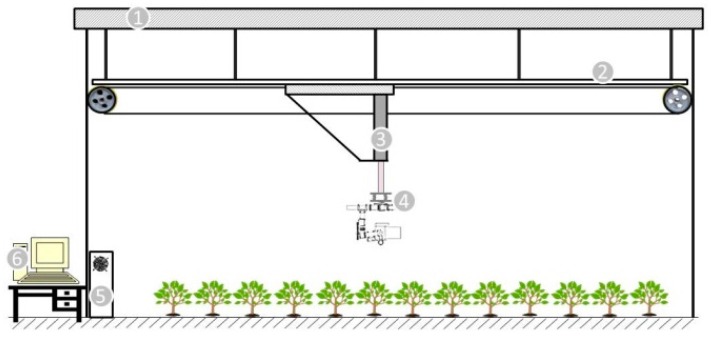
1 is the ceiling; 2 is the guideway made by three 4 m long linear rails; 3 is a vertical actuator; 4 is the gimbal with a camera on it; 5 is the central controller; 6 is a computer with the upper machine software.

The primary purpose of developing this system is to precisely control the sensors’ position and orientation. In remote sensing, POS information is crucial auxiliary information for image georeferencing and rectification [18,29]. A five DOF system including horizontal, vertical, pan, tilt, roll was built. This system can be separated as three parts: machinery part, main controller, computer with upper machine software. The framework of this system is illustrated in Figure 1. A 3-axis gimbal was designed to control the rotational DOFs including pan, roll, and tilt. A stabilizer was mounted on the gimbal to fix the imaging sensor at a certain angle and hold it tight. The altitude data is part of the POS information for further analysis. A linear rail was hoisted under the ceiling for moving the slider and an electrical cylinder was mounted on the plane formed by two sliders. The electrical cylinder was used for vertical position control. Both the linear rail and actuator were driven by servo motors which can precisely control the camera’s position.

A square roller linear guide was selected for the horizontal motion. To obtain highly precise position control, the horizontal rail is required to have high strength and collimation. Linear guides are often used for moving part support and guidance and are commonly seen in high precision mechanical structures like industrial automation equipment [30], PCB printing and precise measurement apparatus. They feature exceptional accuracy and load-bearing capacity.

Wired mode was selected to transmit control orders and feedback information between the gimbal part and the control center. It is hard to choose between wired and wireless method, as the wireless method can save a lot time in arranging cables. The cables can be 15 m long or even longer and the voltage drop is a problem that needs to be addressed. The reason why the wired method was ultimately selected is that the wired method can reduce lag times as much as possible [31]. A CAN bus was used for control order and feedback information transmission. The CAN bus was originally developed for automobile automation control and has evolved to be a reliable communication method. It features unlimited sensing and actuator nodes, simple structure, long transmission distance and high instantaneity, which fits the needs of this project. A CCD camera was mounted on the gimbal and the video was transmitted to the control center for real time monitoring.

**Figure 2:**
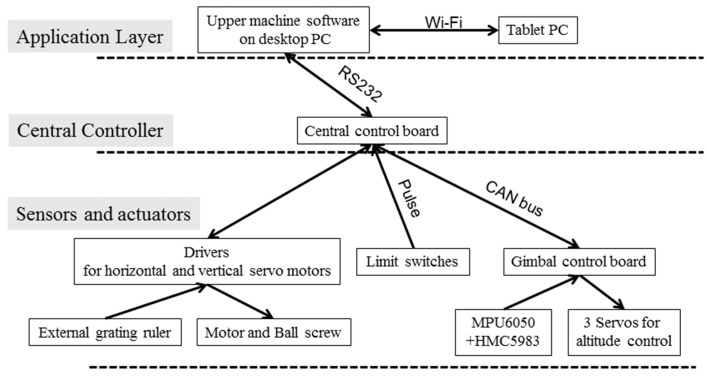
Illustration of the system structure.

The power supply system needs to be carefully designed to avoid signal interference. The power supply types includes: AC 220 V for the horizontal and vertical servo motors, DC 24 V for the control board, 24 V proximity switch, and 11.1 V battery for onboard control, CAN bus cable. Anti-jamming measures are needed to avoid interference between the different voltage cables. The control board connects with the servo motor encoders, limit switches, gimbal and onboard devices as the figure illustrates. The application layer consists of upper machine software for both PC and mobile devices, and a Wi-Fi relay. C++ MFC was selected for GUI development. The software should be able to control the system operation and display how the system is functioning at the same time. Moreover, all data is supposed to be gathered in the upper machine software and stored on disk for further image processing. The system operation covers: respective part control like horizontal movement speed and position control, vertical movement speed and position, angle of pitch-roll-yaw, linked movement, and Wi-Fi transmission setting. The displayed and stored information includes position and altitude of the camera, temperature, illuminance conditions, time, *etc*. System control flow is shown in Figure 2.

### 2.2. Machinery Part

#### 2.2.1. Linear Rail

**Figure 3:**
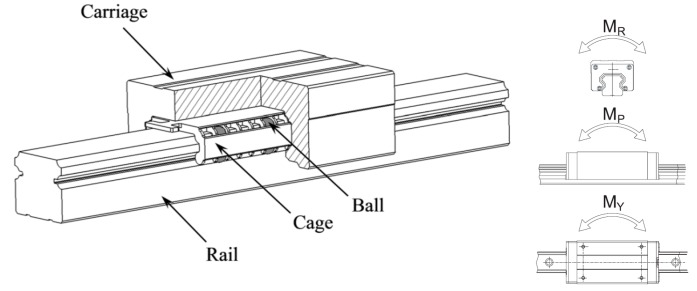
Structure of the slider and its load capacity.

The horizontal guide rail was suspended below the ceiling. To make a 12 m long guide rail, three 4 m long HGH30HA linear rail rolling bearings (Hiwin Technology Corp., Taiwan, China) were mounted on channel steel and joined butt to butt. This linear rail is a square, heavy duty type from the HG series of Hiwin linear rail. The width of the rail is 30 millimeters and the sliders are M8×25 which are designed to sustain 38.74 KN from either pull or press force, 0.88 kN-m static moment from M_R_, 0.92 kN-m from M_P_, 0.92 KN-m from M_Y_. The unique structure illustrated in Figure 3 enables linear rail to have a uniform response against forces from different directions. 

A reference surface was lathed on the base steel channel to mount the rail. All these weighted nearly 300 kg and were hoisted under the ceiling. To reduce inner stress and make the structure stable, the two sliders were screwed on a steel channel and formed a triangle with the vertical actuator. Meanwhile, this structure can also resist instantaneous acceleration when starting and deceleration when stopping. The working principle of the linear guide working principle was illustrated in Figure 3. A Dorna servo motor (Dorna Technology, Jiashan, China) was used to drive the sliders. The working power was 1200 W.

At both ends of the rail, two photoelectric proximity switches were used: one for calibration and the other for position limitation. A NPN normally-closed proximity switch (Roko Technology Ltd., Leqing, China) was used to detect if the slider reach the point. The proximity switches is a very reliable sensor which features quick response, long lifespan and strong anti-interference properties. Its detection distance is 5 mm. The proximity switch was powered by the central controller board and sends pulse signals to the main controller. The position of proximity switches was set to be the horizontal origin point in the system coordinates.

#### 2.2.2. Vertical Actuator

The actuator is an integrated design with the servo motor with a bass screw module. The servo motor used was a Panasonic MSME022G1 AC servo motor 200 W (Panasonic Co. Ltd, Osaka, Japan). An electromagnetic NPN normally closed proximity switch was fixed at the bottom end of the actuator. Its working theory was similar to that of the horizontal one, but it senses the magnetic ring inside the actuator around the guide screw.

#### 2.2.3. Gimbal

A gimbal which can revolve around three DOFs was adopted to simulate the pitch-roll-yaw of sensors’ action on a UAV. 25 mm carbon fiber poles were truncated for the bearing structure, and CNC processed aluminum alloy connecting pieces and light engraved carbon fiber plates were used to join each part together. Four vibration absorber cushions were added. Absorber cushions are commonly used in aerial photography since the vibration generated by motors and the UAV’s attitude adjustment needed to be attenuated to minimize camera shake and avoid ghost phenomena. This system was designed to fit different types of camera, so the servos to drive the gimbal were interchangeable to match the cameras’ weight. For now, three TL100A15 (Tarrot RC Ltd., Wenzhou, China) digital servos were used to change the pitch, roll, yaw angle. Each servo is a combination of a DC motor, control circuit and gear reducer which are encapsulated in a small box. A potentiometer is added to the servo so as to provide closed-loop control to achieve a high precision control effect. These servos were originally used in remote control model planes to control the airfoils and have grown to be effective actuators in industrial automation, especially in the robotics field.

### 2.3. Control Unit

#### 2.3.1. Gimbal Stabilizer

A MPU6050 (Invensense Ltd., Sunnyvale, CA, USA) module and a HMC5983 (Honeywell Ltd., Morristown, NJ, USA) module were used to sense the attitude of the onboard camera. The MPU6050 is a widely used and relatively accurate MEMS six axes motion processing unit with a gyroscope and accelerometers [32]. It has a digital motion processing unit to process raw data from the gyro and accelerometers and outputs processed results (quaternion and attitude) to a central controller via the IIC protocol. The DMP is a unique hardware feature of InvenSense MPU devices which is capable of computing quaternion data from sensor readings, performing device calibration, and also includes application specific features such as pedometer step-counting. The DMP image (firmware) is held in volatile memory on the MPU and needs to be updated to the DMP every time the chip powers up to leverage this functionality. HMC5983 is a temperature compensated three-axis magnetometer. It was used to sense the course angle of the camera. Even though there was magnetic declination between the magnetic south and the geographical south, HMC5983 was to sense the angle between the camera and the self-defined south. The self-defined north-south axis was along the horizontal linear rail. The HMC5983 could sense and output temperature at the same time.

**Figure 4:**
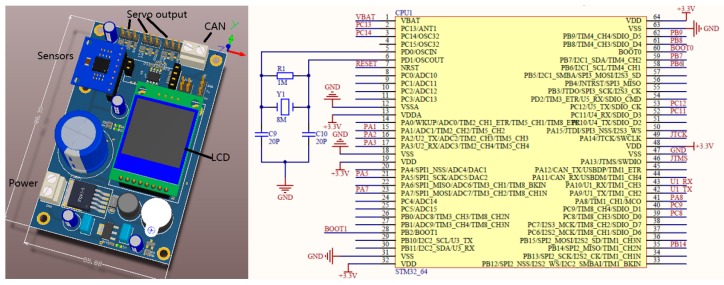
Far-end gimbal controller sketch and wiring diagram.

A control board was used to get attitude data and maintain the gimbal at a certain orientation. The control board’s sketch is shown in Figure 4. A STM32F103RCT6 (STMicroelectronics Ltd., Geneva, Swiss) was to receive attitude control orders from the central controller via CAN-bus and stabilize the gimbal at the assigned attitude. The STM32F103 is an embedded ARM 32-bit Cortex™-M3 CPU with 72 MHz maximum frequency, 256–512 Kbytes of SRAM and 4 -bit timers. It has plenty of interfaces, including CAN, I2C, SPI, UART and USB. An 8 MHz crystal oscillator was matched to generate a time count circuit. The classical PID control method was used as the flow chart illustrates and the control cycle was 10 ms. Compared to commonly used gimbal like the Zenmus (DJI Ltd., Shenzhen, China) and open source gimbal controllers like BGC Russia, PID was used in this case rather than PD control so as to eliminate steady state errors and keep the gimbal at the assigned attitude. The reason why PD is used in commodity gimbals is that they are designed for video shooting and PD is enough to stabilize video frames. A LM2576 regulated power supply was used to transform 5~40V DC to constant 5 V at maximum 3A output. A 5000 mAh Lipo battery was used to power up the gimbal part. The onboard camera is changeable, but for now a Nikon D90 and a Tetracam ADC multispectral camera were used in this system.

**Figure 5:**
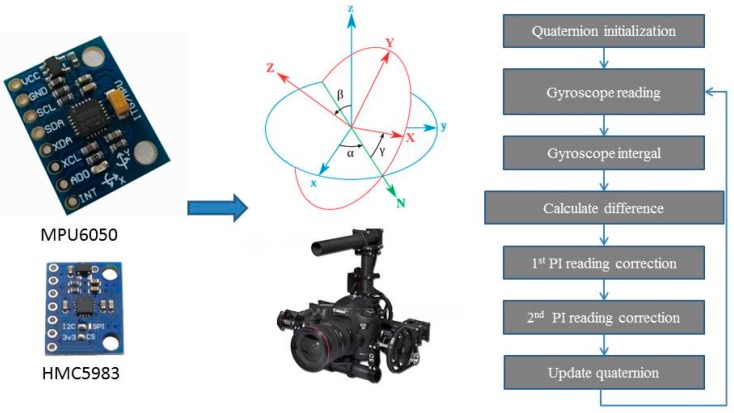
AHRS data fusion steps.

Classical PID was used for the stm32 gimbal stabilizer to control the gimbal altitude using data collected from the MPU6050 and HMC5983 units. The two source datasets were fused for AHRS calculation [33]. The data from the accelerometer, gyroscope and magnetometer can’t be directly used for altitude control. In an AHRS system, altitude change is represented as pitch, roll and yaw which are namely the Euler angle, which is a vector that represents the transform of a rigid body from one state to the next one. If the gyroscope data was completely correct, the altitude control will only need the PID control of the gyroscope but unfortunately all MEMS units don’t have consistent output while the arbitrary environment is changing and the circuit voltage supply has slight fluctuations, so gyroscope data needs gravitational field and geomagnetic field support for correction. PI was used in the AHRS so as to eliminate errors between the gyroscope integral value and accelerometer readings. The working theory of this gimbal was illustrated in Figure 5.

#### 2.3.2. Communications

A CAN bus was used for order communication and working status feedback. The CAN bus protocol was first deployed by the Bosch company on automobiles in early 1980s. It’s a high-speed serial bus protocol which is now widely used in networks consisting of multiple masters [34,35]. All nodes are free to transmit data and orders to the all other nodes inside the control network. Other nodes receive messages and decide whether the message is relevant by checking the message identifier. Concurrent data transmission is solved by message identifier priority as well. Another key feature of the CAN bus is its robustness against errors. The CAN standard only defines three layers namely the object layer, the transfer layer, and the physical layer, while the application layer could be CANopen, DeviceNet, CAN Application Layer (CAL). A CAN-compliant node consists of three parts: the host processor, the CAN controller, and the transceiver. Each controller detects errors and takes appropriate measures to guarantee the consistency and reliability of transmitted data. The CAN bus specification has two versions, namely 2.0A (the original version) and 2.0B (the extended version). The difference lies in the message headers: CAN 2.0A contains 11-bit head identifiers while CAN2.0B contains 29-bit head identifiers. In this research, CAN2.0B was used. The data rate of the CAN bus was set at 57600 bps. The transmitted message structure consisted of the 29 bit identifier, camera attitude data with temperature, data field length, format, and type. The maximum data rate is limited by the bus length. In cases where the transmission distance is less than 40 m, the CAN bus can reach rates as high as 1 Mps. A data rate of 125 kbps would allow a network length up to 500 m. However, it is possible to use bridge devices or repeaters to increase the allowed distance to more than 1 km.

#### 2.3.3. Central Controller

**Figure 6:**
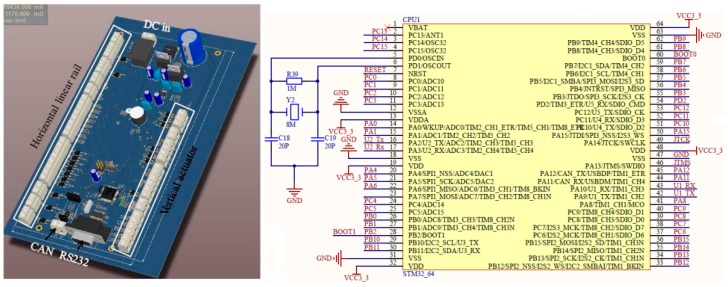
Central controller sketch.

The controller communicates with all parts of the system. Another STM32F103RCT6 with a 8 MHz crystal oscillator was used as the CPU for the central control board. It communicated with upper machine software via a RS232 cable. The cable was adapted to USB via a CH340 serial-to-USB adapter. The control flow of the central controller is illustrated in Figure 6.

### 2.4. Upper Machine Software

**Figure 7:**
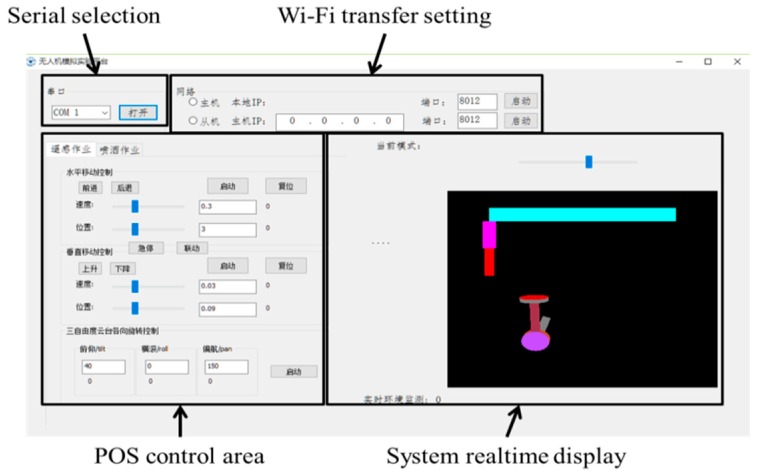
Upper machine software GUI.

The upper machine software was developed as Figure 7 shows using VS2010. It can be run on any Windows PC. Via this software, an operator can easily move the camera to any desired position and orientation and change the order at any time. Moreover, this software was designed to support linked motion which means that the moving part can execute horizontal motion, vertical motion, pitch, roll and yaw at the same time. The animation displays how the system is functioning. Moreover, this software supports Wi-Fi transfer so that we can control the system wirelessly via a tablet PC.

Open Graphics Library (OpenGL) was used to display the movement status of the platform. OpenGL, produced by SGI in July 1992, is a multi-platform, high-performance 3D graphics software development system, that provides a strong design capability in two and three-dimensional graphics. The moving parts include the horizontal movement, vertical movement, pitch angle, roll angle and yaw angle.

### 2.5. Board Sensors

This platform is compatible with a lot of common image sensors, like digital single lens reflex cameras (D90, Nikon Corporation, Tokyo, Japan), multispectral cameras (ADC, Tetracam, Inc., Chatsworth, CA, USA). Besides these, it facilitates testing of some sensors that haven’t been developed for UAV aerial remote sensing like ToF camera (PMD Technology, Siegen, Germany), thermal cameras (Arthereo, Artray, Tokoyo, Japan). If the image sensor has a regular remote trigger connection, the preserved output port of gimbal controller can control the image sensor to take images at the desired position.

## 3. Results

The primary purpose of this system was to develop an easy-to-use platform to precisely control the position and orientation of different types of sensors. The requirements of the system were: high control accuracy of position and orientation, low vibration and shaking. The test consisted of two parts: position and orientation control accuracy test, and vibration and shaking test. It was necessary to take safety into consideration. This mainly consisted of implementing a limit stop mechanical structure and speed output smoothness.

### 3.1. Position and Orientation Control Accuracy Test and Calibration

**Figure 8:**
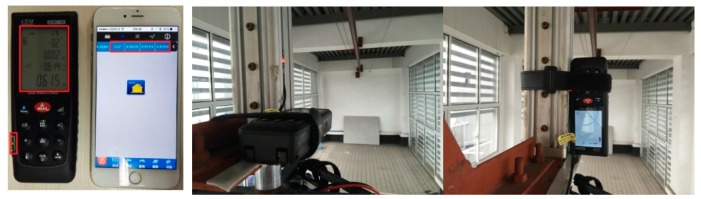
The left photo is the laser distance meter connected with a mobile phone via Bluetooth; the photo on the right shows the laser distance meter installation method.

It was critical to set all original points of the horizontal and vertical and initial direction of pitch-roll-yaw. To precisely control and calibrate the system, it was necessary to know what the control effect is. An iLDM-150 laser distance meter (CEM Ltd., Shenzhen, China) was used to measure the position of the camera. Its measurement range was 0.05~70 m and the precision was ±1.5 mm. It uses 635 nm laser light which is type™II below 1 mW for time of flight distance measurements. The laser distance meter was installed at the end point of the actuator. The horizontal installation error was ±0.1° pitch error and ±0.2° yaw error. The yaw error was calculated via trigonometric function. The vertical installation was checked with a plumb line. The meter was fastened with tape and triggered by mobile phone via a Bluetooth connection. Figure 8 demonstrates how the laser distance meter works and how the measurements were done.

#### 3.1.1. Horizontal Position Accuracy Test

**Figure 9:**
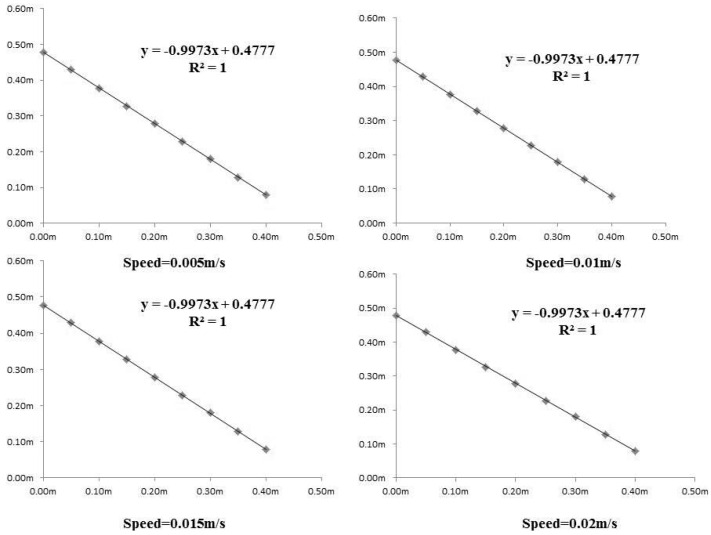
Vertical direction control test results at different speeds.

The wall perpendicular to the linear rail was selected as the reference plane. The system was moving along the horizontal direction at four speed levels: 0.05, 0.1, 0.15 and 0.2 m/s and the test positions were selected at 0.25 m intervals. Every position datapoint was obtained through averaging three repeated tests. The result of 36 position datapoints is shown in Figure 9, where the RMSE at 0.05 m/s speed was 0.002077, at 0.10 m/s it was 0.002848, at 0.15 m/s it was 0.00265 and at 0.2 m/s it was 0.002845. The repeated difference was less than 2 mm.

#### 3.1.2. Vertical Position Accuracy Test

**Figure 10:**
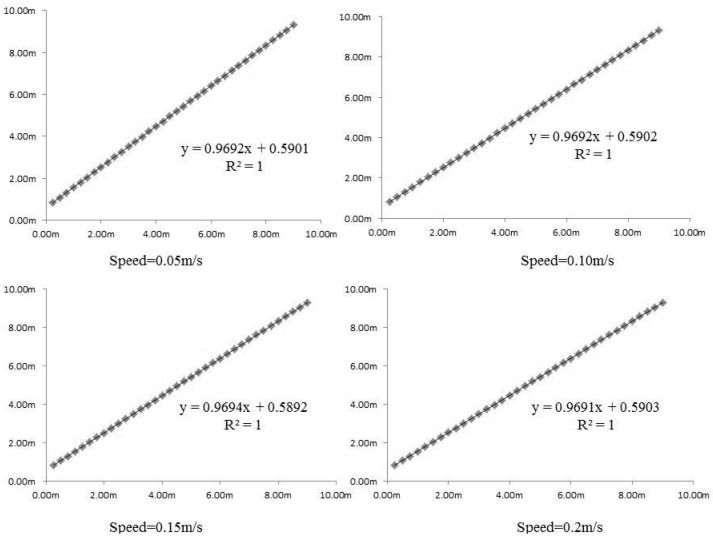
Horizontal direction control test results at different speeds.

The plate mounted at the top of actuator was used as the reference plane. The plate was used to lift the gimbal. The vertical position test was similar to the horizontal test. The system was moving along the vertical direction at four speed levels: 0.05 m/s, 0.10 m/s, 0.15 m/s and 0.20 m/s. The test positions were selected at 5 cm intervals. Every position data point was obtained through averaging of three repeated tests. The result of nine position data points is shown in Figure 10. The x axis is the control order sent from the upper machine software, the Y is the measured distance from the laser distance meter.

### 3.2. Shaking Analysis

**Figure 11:**
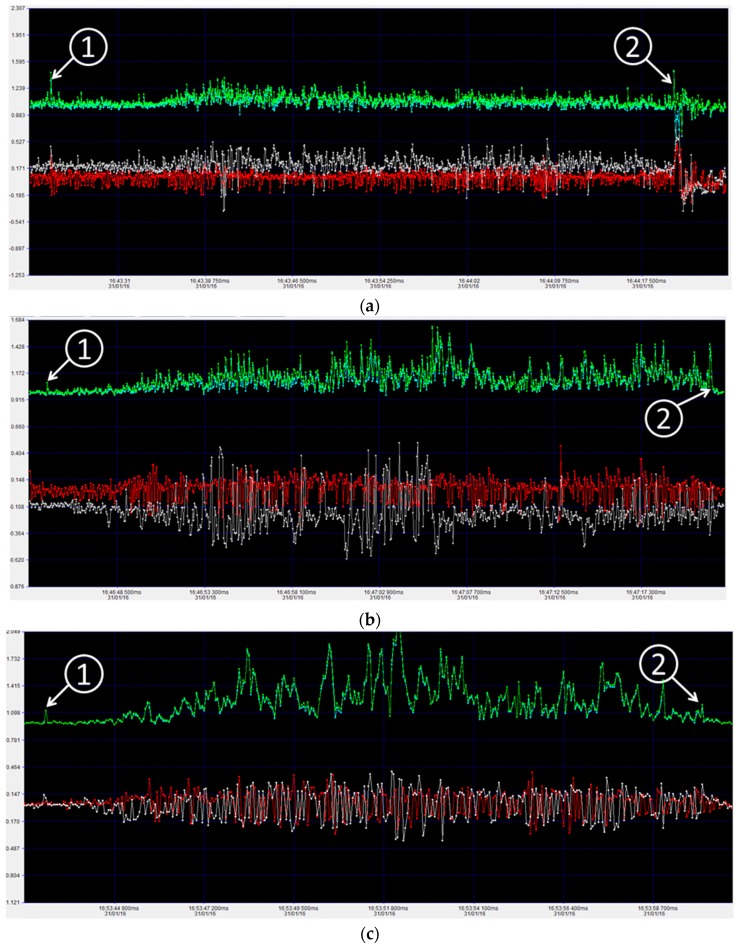
Platform vibration at different moving speed. (**a**) Platform vibration moving at 0.05 m/s; (**b**) Platform vibration moving at 0.10 m/s; (**c**) Platform vibration moving at 0.15 m/s.

System shaking is an inevitable issue in automation systems. It is caused by reasons arising from system installation and operation, for example, the decollimation of the linear guideway may cause shaking, the starting and braking of the system cause impact shaking, any corrosion of the guideway may cause shaking. It’s crucial to know what the shaking is. A shaking and vibration meter (CEM DT-178A, Shenzhen, China) was used to test the shaking of the system while in operation. It is made of a MEMS 3-axis accelerator was is used to test the acceleration. The sampling frequency is 256 Hz, measurement range ±18 g, measurement resolution 0.00625 g. It supports FFT, which can transform time domain signals to frequency domain signals for analysis. The shaking meter was mounted on the gimbal via a magnet. The results when the gimbal moves at 0.05, 0.1, and 0.15 m/s are displayed below. The Figure 11 was fitted to display the shaking and vibration data at the start and stop. The white points and lines represent the X direction acceleration, red means Y direction acceleration, blue means Z direction acceleration, green means summed acceleration. The summed acceleration is 1 g when the vibration meter is kept still.

The figure shows that vibration increases with movement speed. In the 0.15 m/s movement speed test, the peak acceleration is less than 2 g. This means that the system can work smoothly. We can also see that vibration is weak at the start points and lasts for a while then it becomes violent. This may be because the joint between two linear rails increases vibrations. Joints between two linear rails can’t be absolutely smooth in the X, Y and Z direction, and slight variations will result in vibrations. The vibrations will increase along the travel course.

## 4. Discussion and Conclusions

As the result show, the system is highly precise and shows high consistency in repeated tests. The horizontal linear guideway and vertical actuator can resist impacts when starting and stopping. The two servo motors and their drivers make the system achieve that high precision. Since there is a lack of methods to measure the accuracy of the system, it’s considered that the system precision has surpassed the capability of the laser distance meter, especially in the vertical accuracy measurement.

The shaking and vibration results shows that the faster the movement speed is, the more violent the shaking is and the readings are higher at the start and stop ends. Even though a rubber shaking absorber was used between the vertical actuator and gimbal, the shaking is inevitable. This means that the operation speed of the system should work at the required speed.

In general, this system can be used for near ground remote sensing experiments and prepare cameras to be mounted on the UAV. Some theoretical tests can be done on the ground rather than taking the high risk of real UAV flights. Moreover, the position and orientation data can be used for image rectification and referencing just like GPS and orientation data is used for geo-referencing. This system has great potential to perform near ground remote sensing experiments.

## Abbreviations

The following abbreviations are used in this manuscript:

AC	Alternating Current
AHRS	Attitude and Heading Reference System
ARM	Advanced RISC Machine
CCD	Charge Coupled Device
CNC	Computer Numerical Control
DC	Direct current
DMP	Digital Motion Processor
DOF	Degree of Freedom
FAA	Federal Aviation Administration
FC	Flight controller
FFT	Fast Fourier Transform
GUI	Graphical User Interface
MEMS	Micro-electromechanical Systems
PCB	Printed circuit board
PID	Proportion Integration Differentiation
POS	Position and Orientation System
PRI	Photochemical Reflectance Index
RC	Remote control
SPI	Serial Peripheral Interface
SRAM	Static Random Access Memory
UART	Universal Asynchronous Receiver/Transmitter
UAV	Unmanned Aerial vehicle
